# Elucidating drivers of oral epithelial dysplasia formation and malignant transformation to cancer using RNAseq

**DOI:** 10.18632/oncotarget.5529

**Published:** 2015-10-19

**Authors:** Caroline Conway, Jennifer L. Graham, Preetha Chengot, Catherine Daly, Rebecca Chalkley, Lisa Ross, Alastair Droop, Pamela Rabbitts, Lucy F. Stead

**Affiliations:** ^1^ Precancer Genomics, Leeds Institute of Cancer and Pathology, Wellcome Trust Brenner Building, St James's University Hospital, Leeds, LS9 7TF, UK; ^2^ Stratified Medicine (Oncology), School of Biomedical Sciences, University of Ulster, Coleraine, Co. Londonderry, BT52 1SA, UK

**Keywords:** RNAseq, oral squamous cell carcinoma, OSCC, dysplasia, non-coding

## Abstract

Oral squamous cell carcinoma (OSCC) is a prevalent cancer with poor prognosis. Most OSCC progresses via a non-malignant stage called dysplasia. Effective treatment of dysplasia prior to potential malignant transformation is an unmet clinical need. To identify markers of early disease, we performed RNA sequencing of 19 matched HPV negative patient trios: normal oral mucosa, dysplasia and associated OSCC. We performed differential gene expression, principal component and correlated gene network analysis using these data. We found differences in the immune cell signatures present at different disease stages and were able to distinguish early events in pathogenesis, such as upregulation of many HOX genes, from later events, such as down-regulation of adherens junctions. We herein highlight novel coding and non-coding candidates for involvement in oral dysplasia development and malignant transformation, and speculate on how our findings may guide further translational research into the treatment of oral dysplasia.

## INTRODUCTION

Oral squamous cell carcinoma (OSCC) is the 6^th^ most prevalent cancer worldwide [1] with a 5-year survival rate of just 50%. Malignancy drivers for the 95% of OSCC that is HPV-negative are sorely needed as these patients have the worst prognosis. OSCC is proposed to occur via a stepwise model whereby genetic abnormalities accumulate, resulting in abnormal lesions called dysplasia, with higher risk of malignant transformation into OSCC than histologically normal oral mucosa [2].

To further understand the development and malignant transformation of oral dysplasia at the cellular level, we have characterised the transcriptomes of matched normal oral mucosa, oral dysplasia and associated OSCC in 19 patients in unprecedented detail, using high coverage strand-specific RNA sequencing (RNAseq) that captures information about both coding and non-coding RNA (ncRNA > 200bp). Functional ncRNAs are now known to be associated with numerous diseases, including cancer, where there is evidence of their involvement throughout all stages of development and progression [3].

Ours is the largest study of matched non-HPV infected patient trios, where all dysplasias are associated with OSCC, that has been performed to date, and the first to include long ncRNAs.

## RESULTS

**Figure 1:**
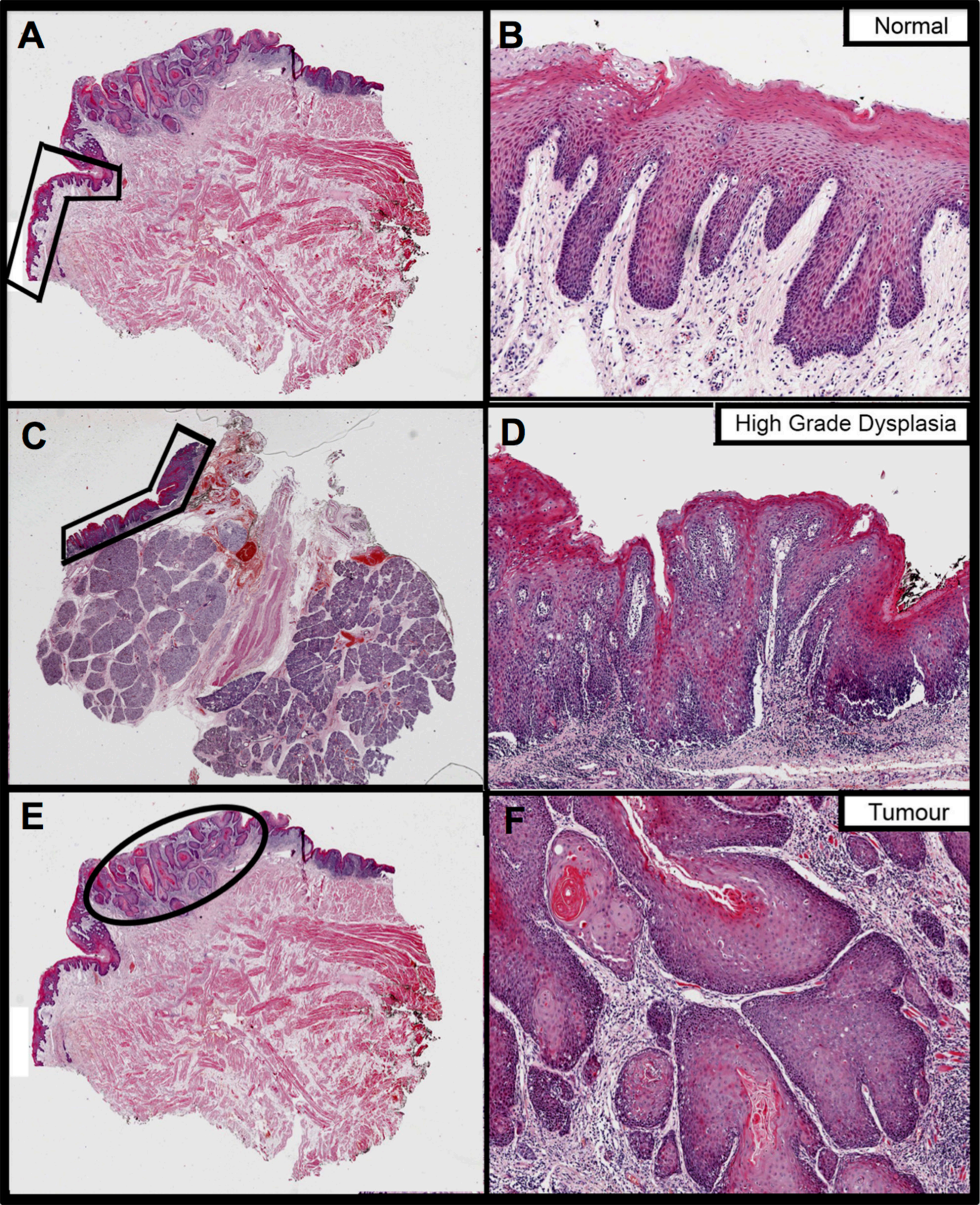
The fixed sections that were H&E stained and annotated to guide RNA extraction from a single patient (ID PG063) in this study Images on the right are magnifications of the areas annotated on the left, to better show histology. Images **A.** and **B.** pertain to the normal oral mucosa sample, images **C.** and **D.** to dysplasia and images **E.** and **F.** to tumour. Normal and tumour were extracted from the same block whereas dysplasia is from a different block from the same surgery.

19 HPV-negative patients had RNA extracted and sequenced from three samples: normal oral mucosa (N), dysplasia (D) and OSCC tumour (T). Figure 1 gives an example of a sample trio from a single patient. Clinical information, library preparation and sequencing metrics are in Supplementary Table S1, Supplementary Table S2 and Supplementary Results.

### Differentially expressed genes (DEGs)

**Figure 2:**
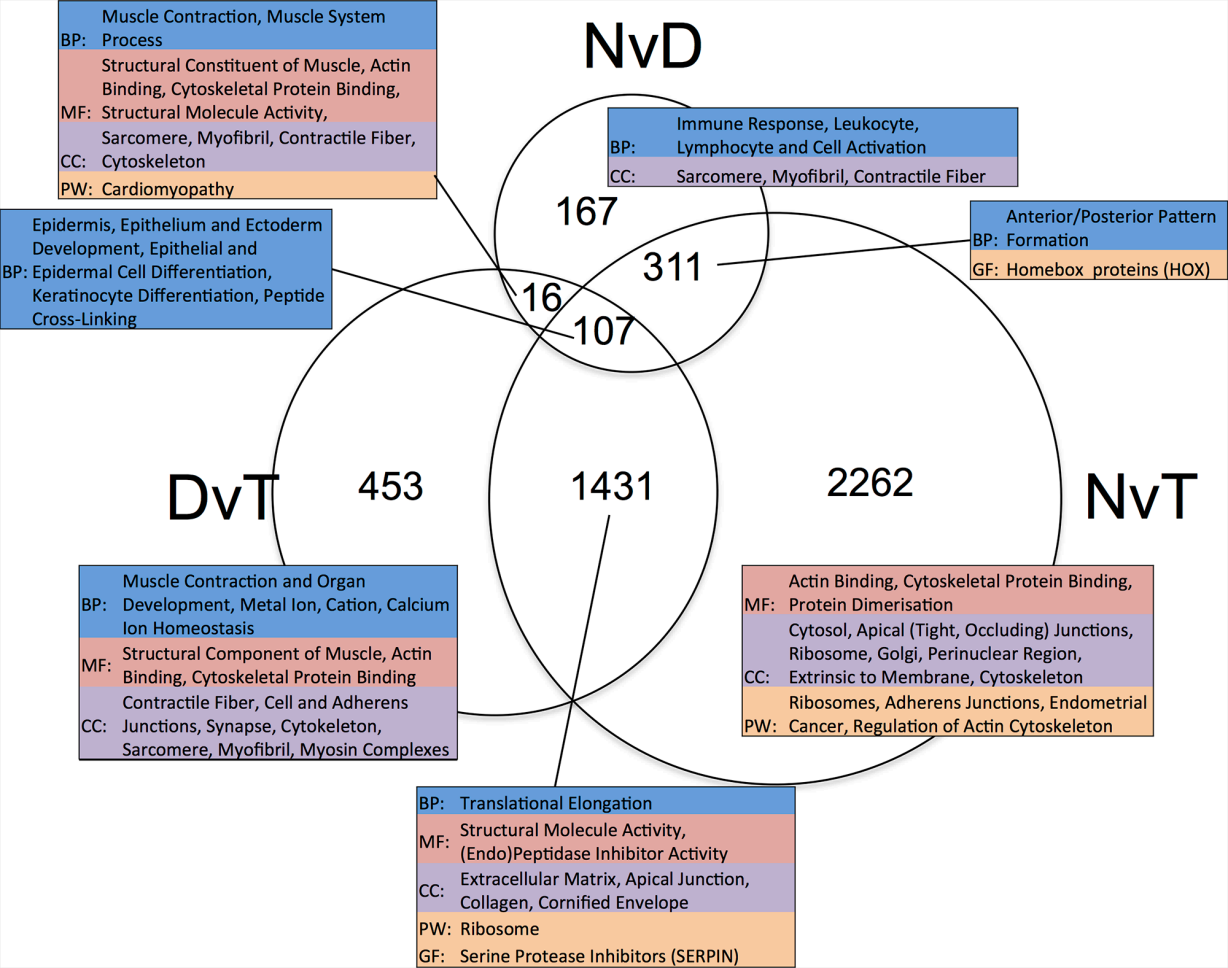
Venn diagram showing the overlap in lists of differentially expressed genes ascertained per pairwise, matched-sample comparison of our dataset The numeric labels indicate the number of genes in each set. The tables highlight the significant (*p* < 0.05) functional enrichment within each subset according to gene ontology terms (BP: Biological Process, CC: Cellular Component and MF: Molecular Function), pathway (PW) analysis using Biocarta, KEGG and Panther, and gene family (GF) enrichment according to Panther. NvD: Normal versus Dysplasia. DvT: Dysplasia versus Tumour. NvT: Normal versus Tumour.

We performed three pairwise, matched-sample comparisons: Normal versus Dysplasia (NvD), Dysplasia versus Tumour (DvT) and Normal versus Tumour (NvT). Per gene results are given in Supplementary Table S3 and numbers of significantly differentially expressed genes (DEGs) in Supplementary Table S4. The overlap in DEGs for each pairwise comparison is shown in Figure 2. We inspected the functional enrichment within the resulting subsections, each with a different biological interpretation, independently (Figure 2). Assuming the three groups represent disease progression i.e. normal epithelium become dysplastic, and dysplasia malignantly transforms to tumour, we can inspect our DEGs in terms of early and late events [2].

### Genes associated with the formation of dysplasia

**Figure 3:**
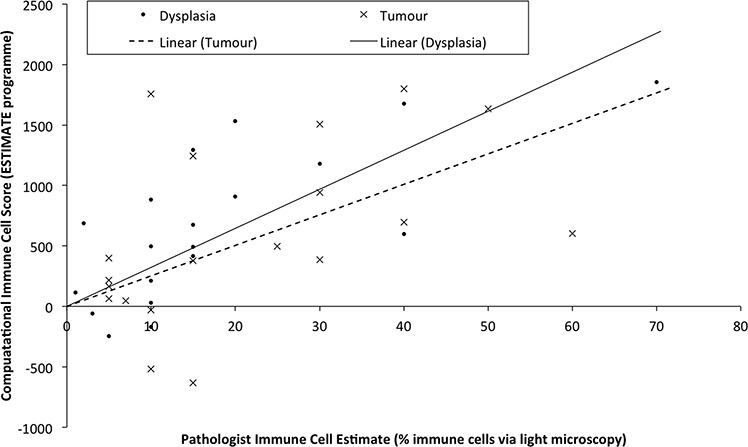
Samples are plotted according to the pathologist estimates of the percentage of immune cells within the macrodissected FFPE tissue (x-axis) versus the immune cell score derived computationally from the transcriptional profile Linear regression lines for each tissue type are drawn separately as Linear (Tumour) or Linear (Dysplasia).

**Figure 4:**
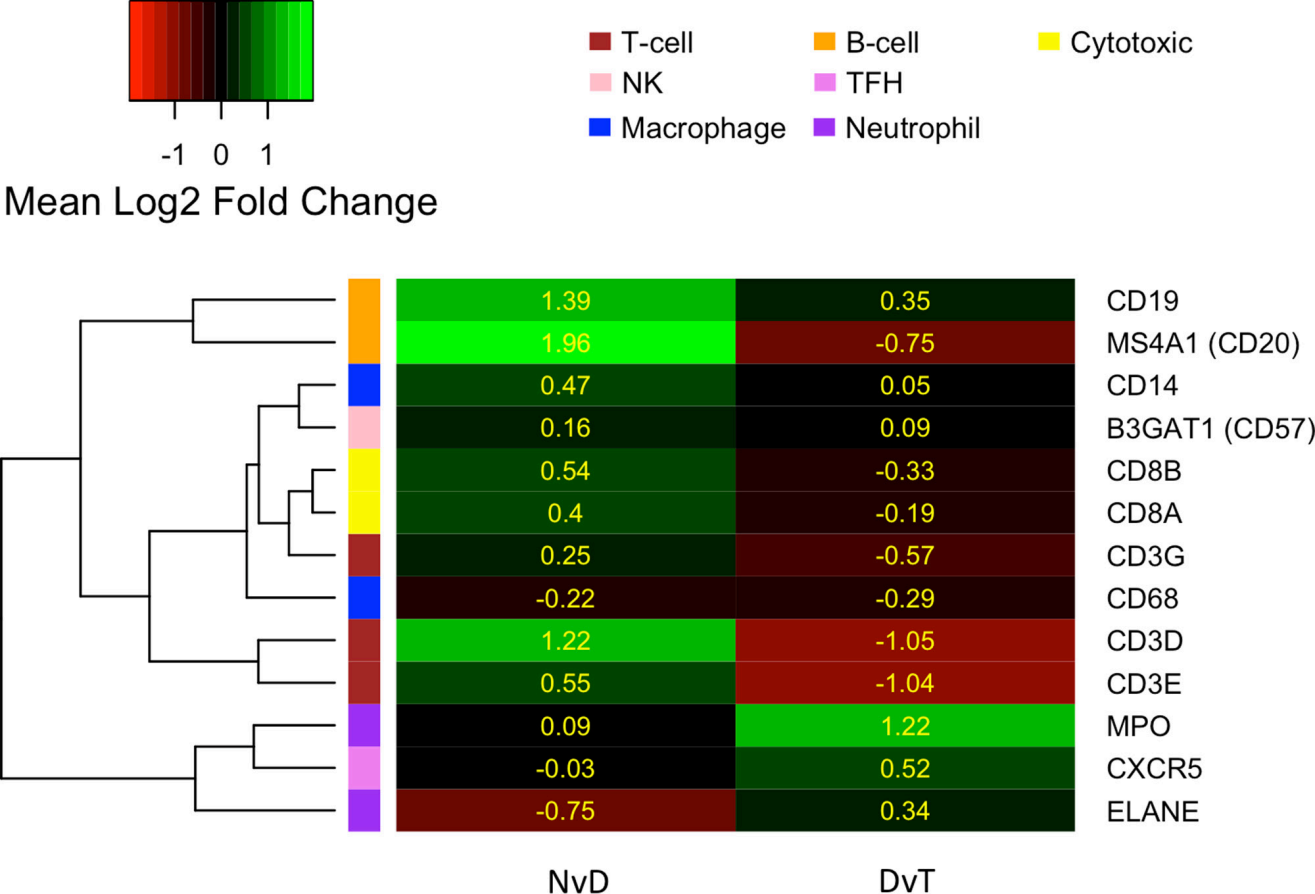
Heatmap indicating the average log_2_ fold change in expression (yellow values) of commonly used immunohistochemical markers for different immune cell types, as per the left hand colour key and top-right legend Marker genes (row labels) are clustered according to their expression. NvD: Normal versus Dysplasia. DvT: Dysplasia versus Tumour

Genes that are DE between NvD, but not DvT, are dysregulated early, as a cause or consequence of the initial development of abnormal, non-malignant cells. This subset contains 478 genes, significantly enriched for immune response (p.adj: 4.3 × 10^−4^) and extracellular location (p.adj: 5.6 × 10^−3^). Within these is a subset, 167 genes, DE NvD but not NvT (Figure 2). These are still enriched in immune response (p.adj: 4.4 × 10^−4^) but leukocyte (p.adj: 2.2 × 10^−2^) and lymphocyte (p.adj: 2.6 × 10^−2^) activation are specifically significant. This is interesting because immune responses are not enriched DvT, despite the pathologist estimates of infiltrating immune cells being higher in tumour than dysplasia in two thirds of our patients (Supplementary Table S5). To inspect the association between immune cell transcriptional signals and pathologist estimates, we applied a programme for Estimation of STromal and Immune cells in MAlignant Tumours using Expression data (ESTIMATE) [4]. This program uses immune cell specific gene signatures to score/predict the amount of immune cells within the tissue. Results are given in Figure 3 and Supplementary Table S5. The pathologist immune cell percentage significantly correlates with ESTIMATE immune score for both tissue types but both the correlation coefficient, r, and level of significance is greater for dysplasia versus tumour (dysplasia r:0.72 and p:0.0005 versus tumour r:0.49 and p:0.03). This indicates that, despite there mostly being more immune cells at the tumour stage, the per-immune cell transcriptional signals are stronger in dysplasia. We then used the approach described in Bindea et al. [5] to inspect the ‘immunome’ i.e. which specific types of immune cells are involved during different disease stages (NvD compared to DvT). Our results (Table 1) indicate that cytotoxic effector immune cells infiltrate the dysplasia tissue compared to normal tissue, whereas tumour is enriched in inflammatory immune cells compared with dysplasia. The average fold changes in expression of classical immunohistochemical markers for these cell types are also given in Figure 4. To validate these findings we then inspected the differential expression of immunome genes, NvD and DvT, in an independent cohort (dataset GSE30784) of unmatched N (45), D (17) and T (167) samples. A significant (Fisher test, *p* < 0.05) number of (i) immature dendritic cell and mast cell genes were DE NvD, with an average 23% and 18% increase in expression respectively, and (ii) mast cell and macrophages were DE DvT with an average 44% decrease and 100% increase in expression respectively (Supplementary Table S6). Mast cells are cytotoxic effector cells within the oral cavity [6]. Despite the unmatched nature of this dataset, which negates the ability to account for idiosyncrasies of an individual's immune system, this result also indicates an increase in cytotoxic cells at the stage of dysplasia and a decrease in these cells, with concomitant increase in inflammatory cells, in the tumour tissue. We also applied an approach described in [7] whereby an RNA-seq based metric of immune cytolytic activity was devised and applied to numerous cancer samples. This metric, CYT, is calculated as the geometric mean of two key cytolytic effectors: granyme A: *GZMA*, and perforin: *PRF1.* Owing to the matched nature of our data we were able to trace the change in CYT score between samples within each patient (Supplementary Figure S1), which again indicated a more pronounced increase in cytolytic activity between N and D (0.72 ± 0.25 s.e.m) than between N and T (−0.01 ± 0.41 s.e.m).

**Table 1:** Immune-specific cell-types (as per analysis in Bindea et al. [5]) for which a significant number of genes are up-regulated, and the genes up-regulated therein

NvD		DvT	
Cell-type	Genes	Cell-type	Genes
aDC	*LAMP3, OAS3*	Macrophages	*MARCO, BCAT1, FN1, MSR1, SULT1C2, PCOLCE2, SCG5*
B-cells	*BLK, KIAA0125, MS4A1, SCN3A, IGKC, SLC15A2, SPIB*	Neutrophils	*BST1, DYSF, FPR1, MME*
Cytotoxic cells	*APBA2, GNLY, GZMA, NKG7, RUNX3*	Tgd	*C1orf61, FEZ1*
NK CD56 dim cells	*IL21R, GZMB*	Th1 cells	*DGKI, DOK5, EGFL6, GGT1*
T-cells	*LCK, CD2, CD28, CD3D, CD3E*, *CD6, SH2D1A*	Th2 cells	*MB, ADCY1, PTGIS, ANK1, PHEX*
TFH	*CXCL13, SIRPG, TOX*		
Th1 cells	*BST2, CD38, CTLA4*		

Abbreviations: NvD: Normal versus Dysplasia. DvT: Dysplasia versus Tumour. aDC: activated Dendritic Cell. TFH: T Follicular Helper cell. Th1/2: T-helper 1/2 cell. Tgd: T gamma delta cell.

The 311 genes DE NvD and NvT but not DvT are significantly enriched for anterior/posterior pattern formation, driven by numerous homeobox (HOX) genes. Humans have 39 HOX genes, 26 being expressed in our data. Of these, a significant number are DE both NvD (7 genes, Fisher p: 6.8 × 10^−6^) and NvT (13 genes, Fisher p: 0.0015) and several ncRNA genes expressed antisense to the HOX clusters are DE NvT. *HOTAIRM1*, transcribed antisense to the HOXA cluster and believed to have a role in myeloid cell differentiation, is also DE NvD [8]. Only one HOX gene, *HOXB7*, is DE DvT: no more than is expected by chance. In every case where a HOX, or antisense HOX, gene is DE it is upregulated (Table 2). We ruled out copy number/gene dosage as the cause of these results by inspecting all expressed genes 2 Mb up and downstream of the 4 HOX clusters and finding no similar significant upregulation therein.

**Table 2:** Log_2_ fold change values (Log2FC) and multiple-testing adjusted *p*-values for HOX genes and antisense HOX genes that are significantly (values in red bold) differentially expressed in at least one pairwise comparison at the 1% threshold

GeneName	GeneType	NvD		DvT		NvT	
		Log2FC	Adjusted p	Log2FC	Adjusted p	Log2FC	Adjusted p
*HOXA1*	protein-coding	1.66	**7.04E-03**	−0.26	8.26E-01	1.62	**8.51E-03**
*HOXA4*	protein-coding	1.65	1.36E-01	0.24	1.00E+00	2.33	**3.73E-03**
*HOXA7*	protein-coding	1.93	**9.69E-03**	0.23	9.18E-01	2.23	**8.15E-04**
*HOXA10*	protein-coding	2.21	**3.25E-04**	0.06	1.00E+00	2.24	**3.78E-05**
*HOXB6*	protein-coding	1.17	2.25E-01	0.81	1.15E-02	2.07	**4.00E-04**
*HOXB7*	protein-coding	0.81	5.86E-02	1.05	**7.31E-03**	1.86	**9.84E-06**
*HOXC4*	protein-coding	1.69	**7.58E-04**	0.41	3.06E-01	2.10	**8.13E-08**
*HOXC6*	protein-coding	2.90	**1.53E-08**	0.48	3.44E-01	3.37	**5.54E-14**
*HOXC9*	protein-coding	2.94	**7.15E-05**	1.01	2.09E-02	3.85	**5.18E-11**
*HOXC10*	protein-coding	1.36	1.96E-01	0.51	5.57E-01	2.14	**6.24E-03**
*HOXC13*	protein-coding	1.18	3.63E-02	0.28	7.35E-01	1.38	**2.35E-03**
*HOXD9*	protein-coding	0.88	2.21E-01	0.52	3.33E-01	1.33	**3.21E-03**
*HOXD10*	protein-coding	2.62	**1.03E-08**	−0.18	1.00E+00	2.65	**7.18E-08**
*HOXC-AS5*	antisense	1.76	1.35E-02	0.46	3.66E-01	2.28	**1.05E-04**
*HOTAIRM1*	antisense	1.06	**2.06E-03**	0.19	8.48E-01	1.25	**2.70E-04**
*HOXD-AS2*	antisense	0.85	9.79E-02	0.36	4.77E-01	1.16	**3.63E-04**
*HOXA-AS2*	antisense	0.92	4.05E-02	0.33	5.44E-01	1.16	**4.90E-03**

Abbreviations: NvD: Normal versus Dysplasia. DvT: Dysplasia versus Tumour. NvT: Normal versus Tumour.

**Figure 5:**
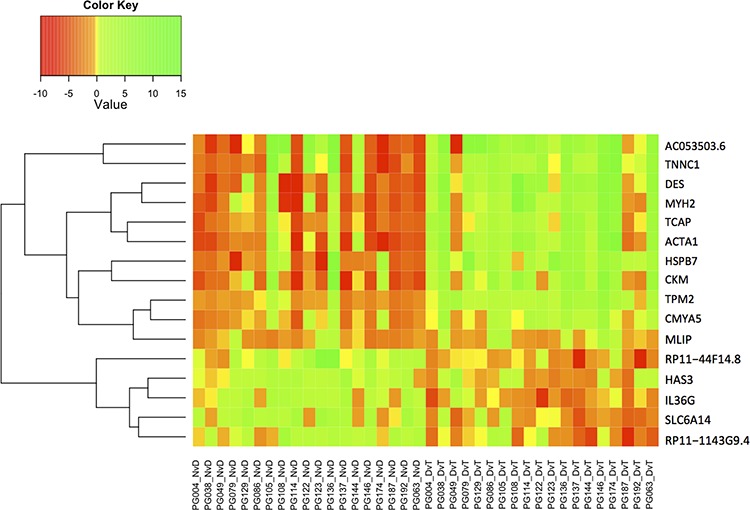
Heatmap indicating log_2_ fold change (Value) for the only 16 genes that are differentially expressed in both the NvD and DvT, but not the NvT pairwise comparison NvD: Normal versus Dysplasia. DvT: Dysplasia versus Tumour. NvT: Normal versus Tumour.

Genes that are significantly DE NvD and DvT, but not NvT must be dysregulated in different directions during the stages of disease progression. Figure 5 is a heatmap of the 16 genes that are significantly DE NvD and DvT, but not NvT, showing two clear groups: genes upregulated NvD then downregulated DvT, and a larger group dysregulated vice versa. We note the presence of *IL36G* within this 16-gene subset. As shown in Figure 5 and Supplementary Figure S2, this gene, Interleukin-36 gamma, is almost wholly upregulated NvD and downregulated DvT resulting in it not being DE in the NvT comparison (p.adj: 0.70).

### Genes associated with malignant transformation of dysplasia

Genes DE DvT but not NvD are dysregulated later in the pathological process, as a cause or consequence of malignant transformation of the tissue, and are functionally enriched in muscle contraction, actin-binding and cytoskeletal protein-binding. We noted that apical or adherens junctions (the latter form part of the apical junction complex) are highlighted in all subsets of genes that are not DE NvD (Figure 2). We observed that the genes encoding the key components of adherens junctions in both normal and cancerous epithelial cells (E-cadherin: *CDH1*, β-catenin: *CTNNB1*, α-catenin: *CTNNA1*, p120: *CTNND1* and junction plakoglobin: *JUP*) are all significantly down-regulated NvT, except F-actin (*ACTB*) [9]. None were also significantly altered NvD but all except *CDH1* were significantly downregulated DvT at the 10% threshold. Thus, expression of adherens junction components is decreased after dysplasia formation, indicating a potential role in malignant transformation.

### Genes that are consistently altered throughout disease progression

Genes DE in all comparisons are consistently dysregulated throughout OSCC development. These 107 are enriched for epidermal and epithelial cell differentiation, and keratinocyte differentiation specifically (Supplementary Table S7).

### Genes that best distinguish normal, dysplasia and tumour cells

**Figure 6:**
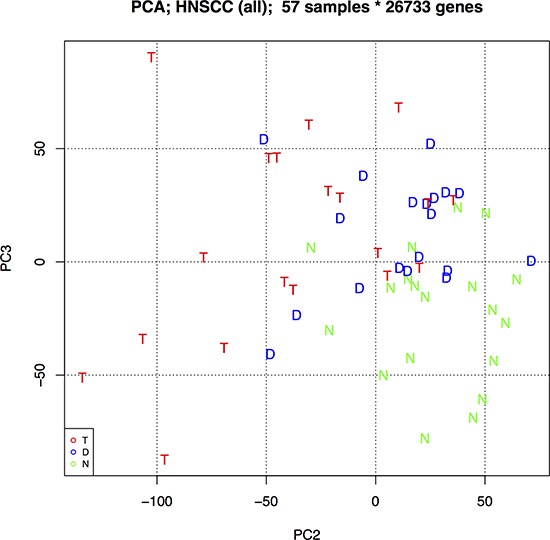
Principal component analysis (PCA) biplot of PCs 2 and 3 using all protein-coding and non-coding genes This biplot best separates the three oral sample groups: N – Normal epithelia, D – Dysplasia, and T – Tumour.

To investigate whether some genes are more informative for separating samples into their associated groups (N, D or T) we performed Principal Component Analysis (PCA), first using just the expressed protein-coding genes. Biplots of each combination of PCs 1 to 10 did not show any visual separation of groups (Supplementary Figure S3A). However, repeating the analysis including expressed non-coding genes (Supplementary Figure S3B) resulted in an evident separation of normal and tumour samples using PCs 2 and 3, with dysplasia samples overlapping on both sides (Figure 6). Using the average magnitude of weighting for each gene across PC2 and PC3 as a gene-ranking metric, the top 5% (Supplementary Table S8) included 904 protein-coding and 162 non-coding genes, were enriched in actin filament-based processes, actin cytoskeleton organization, cytoskeletal and actin binding (p.adj < 0.01).

### Network analysis

**Figure 7:**
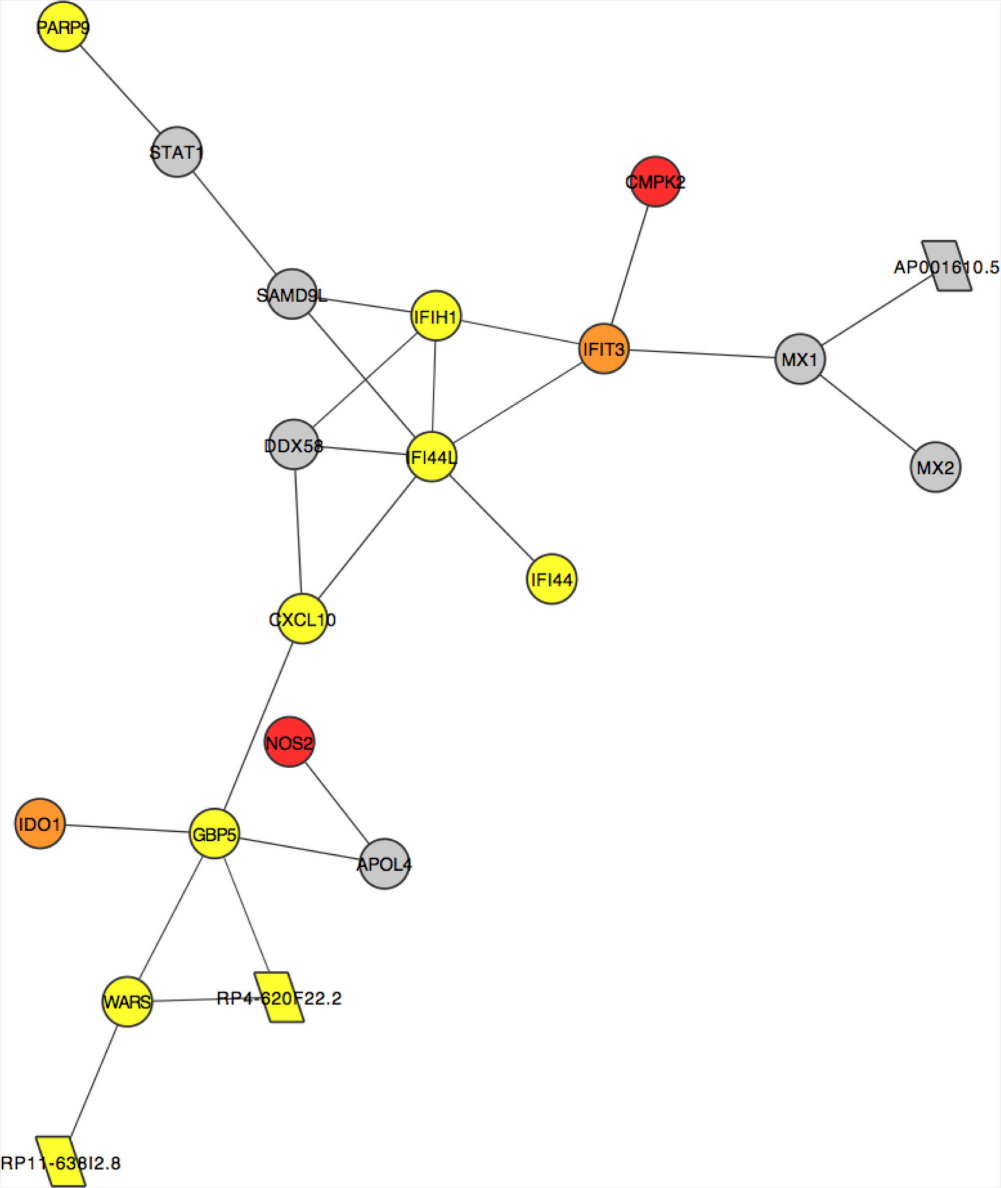
A subcluster of genes that are significantly positively correlated (correlation coefficient >0.95) across all samples in our data Each node represents the labelled gene. Circular nodes indicate protein-coding genes. Parallelograms denote antisense genes. Colours indicate differential expression (DE) or lack thereof (grey) in our pairwise comparisons: yellow genes are DE NvD only, orange genes are DE NvD and NvT and red genes are DE NvD, DvT and NvT.

All of the above analyses have highlighted non-coding genes (Supplementary Table S3 and Supplementary Table S8) that may have a role in dysplasia formation and malignant transformation. However, there is a dearth of functional information about the majority of non-coding transcripts. To prioritise such transcripts as carcinogenic candidates, we created an expression correlation matrix for all (coding and non-coding) expressed genes in all samples. Genes with similar expression profiles likely have similar, or complementary, roles as part of larger gene regulatory networks [10]. Non-coding RNAs of interest can, thus, be inspected within the networks of significantly correlated genes and functional relevance inferred via a ‘guilt by association’ approach as described in Guttman et al, 2009 [11] (Supplementary Figure S4). Subnetworks with particular features can then be inspected as in Figure 7 where a cluster of 20 significantly positively correlated genes are mostly dysregulated early in the pathological process i.e. they are DE in the NvD comparison only (yellow nodes) or both NvD and NvT (orange nodes). Supplementary Table S9 describes these genes, which are significantly enriched for viral response, defense response and immune response. Three antisense non-coding genes in this network, indicated by parallelograms in Figure 7, are each positively correlated with the protein-coding (circular nodes) gene to which it is antisense (Supplementary Table S9). Another subcluster contained several brown nodes (genes DE in all three pairwise comparisons), one of which denotes a lincRNA: *RP11–351J23.1* (Supplementary Figure S5). The genes in this subcluster are significantly enriched for the biological processes of epithelial development and epithelial cell differentiation, and are all downregulated in the comparisons for which they are DE.

## DISCUSSION

A potential confounder to our study is that the common recurrence of OSCC post-surgery, and posited theory of field cancerisation, result in debate regarding whether ‘normal’ oral tissue in OSCC patients is truly free from molecular alteration, and/or whether sufficient margins are taken during surgery to enable excised samples to actually contain ‘normal’ tissue [12]. Whilst we sampled from pathologist-confirmed histologically normal tissue as far from the diseased tissue as physically possible, we cannot rule out that the very nature of its origination in a patient with OSCC may mean that our ‘normal’ tissue differs in some fundamental way from that in cancer free patients. Systematic reviews aimed at consolidating the literature on OSCC transcriptomes have revealed the heterogeneity in reported expression profiles, caused by both technical and biological phenomena [13]. Despite this, the genes highlighted as DEGs in our study have a 91% overlap with those listed in a systematic review of 41 previous NvT studies in OSCC, validating our results and showing at least that the confounding issue of ‘normal’ tissue equally affects the wider body of literature on OSCC gene expression studies like ours [13]. We also note that dysplasias do not always represent an earlier stage of an associated OSCC, but in such cases our study design still investigates changes in transcriptional profiles between non-malignant lesions and invasive carcinoma in the same biological background, increasing the power to detect pathophysiologically relevant alterations [14].

### The immune system responds to dysplasia development

Our results show that immune response genes are dysregulated early, during the development of dysplasia, as corroborated elsewhere [15, 16]. Furthermore, a significant increased in transcriptional signals specifically from cytotoxic effector cells were evident in the dysplasia compared to matched normal (Table 1). Despite attempts to macrodissect regions of high purity normal, dysplastic or tumour tissue, infiltrating immune cells will have inevitably been included during sampling. The more infiltrative these cells are, the harder to dissect around them and the more likely we are to observe their transcriptional profiles. In general the level of infiltration is observed to be greater, under histopathological examination, at the tumour than the dysplastic stage so we may expect stronger immune cell transcriptional profiles in the former. However, we cannot foresee that our sampling approach would produce a bias towards a specific cell type signature unless that cell is actually more present at that disease stage. Analysis of an independent cohort of unmatched N, D and T samples (from microarray study GSE30784) also supported this finding, despite the study design negating our ability to investigate changes in immune response throughout disease development within individual patients, where such findings will become more apparent. The involvement of cytotoxic cells at this early stage supports the concept of cancer immunosurveillance, which postulates that the immune system acts to identify and remove abnormal cells, such as dysplasia [17]. A significant upregulation in macrophage- and neutrophil-specific gene expression is observed between matched dysplasia and tumour, and of macrophage genes DvT in the unmatched validation cohort, indicating the addition of inflammatory immune cells at the tumour stage. These could be the result of a more sustained immune response to the tumour. However, tumour-associated macrophages and neutrophils have also been shown to play a role in creating an immune-suppressive tumour microenvironment in many cancers including head and neck cancer [18, 19]. It is now believed that immunosurveillance is the first of a three-step process referred to as cancer immunoediting, in which abnormal cells are first eliminated, as indicated above, but with the possibility that clonal evolution results in cells able to survive immune attack and, following immune evasion, begin aberrantly proliferating [17]. An interesting hypothesis relating to OSCC, based on the concept of immunoediting, is that malignant transformation occurs in those dysplasia cells able to evade immune attack. Our experimental design cannot be used to test this hypothesis but previous studies in head and neck cancer have revealed immune suppression in tumour regions, and mouse models of oral carcinogenesis suggests that the premalignancy microenvironment is indeed more immune stimulatory than the OSCC environment [20–22]. We also did some preliminary testing of this hypothesis using an additional independent cohort (dataset GSE26549) of 86 oral dysplasias with clinical follow up over median 6.08 years [23]. The genes upregulated in the 51 dysplasia samples from patients that did not develop OSCC (non-progressive) versus 35 that did (progressive) were significantly enriched for genes (22 genes, FDR = 5.16 × 10^−5^, Supplementary Table S10) switched on in stimulated versus naive T cells (MSigDB C7: Nick Haining Lab [DFCI]). This does indicate that dysplasias with a heightened immune response are less likely to undergo malignant transformation.

Genes significantly upregulated NvD and downregulated DvT are candidate malignancy drivers and include *IL36G* as the only immune response gene (Figure 5 and Supplementary Figure S2). This gene, interleukin-36 gamma, is expressed in keratinocytes and the encoded protein may act, besides interferon gamma, as key signaling molecule in immunopathology as part of the local inflammatory response in epithelial cells [24, 25].

### HOX genes as candidate drivers of dysplasia formation and progression

HOX genes all encode transcription factors (TFs) with a conserved homeodomain (HD). First identified for their involvement in spatial development along the anteroposterior axis, they are now known to also participate in maintenance of tissue architecture throughout an organism's lifetime [26]. Dysregulation of HOX genes is common in cancer and has been associated with oncogenesis and acquisition of a stem cell phenotype [27]. In agreement with our NvT results, HOX genes have been observed to be consistently upregulated in OSCC compared with normal oral mucosa [28, 29]. Our results further indicate that the majority of HOX upregulation occurs specifically during the development of dysplasia. We also found that a significant number of HOX genes (12 genes, FDR = 0.03) are upregulated in dysplasias that progress to OSCC versus those that do not (independent dataset GSE26549) further implying that HOX expression is associated with malignant transformation [23].

Long ncRNAs expressed antisense to HOX gene clusters have been shown to act as master regulators and also become dysregulated during cancer [30–32]. Our approach highlighted several antisense HOX transcripts as DE NvT and one, *HOTAIRM1*, is also significantly upregulated NvD (Table 2). These are worthy of further functional characterization in relation to the development and progression of OSCC.

### Malignancy is associated with an invasive phenotype

Genes dysregulated later, during malignant transformation of dysplasia, are enriched in functional terms associated with the extracellular matrix (ECM) and apical or adherens junctions (Figure 2). Dysregulation of genes involved in ECM degradation or remodeling has been implicated in OSCC progression previously and associated with the ability of cells to begin invading through the basement membrane, as is characteristic of malignant transformation [33, 34]. Apical junction complex (AJC) are epithelial cell-cell adhesion structures responsible for cell polarity and maintenance of tissue structure [35]. Our data shows a down-regulation of AJC components, most evident DvT. Overexpression of these components can inhibit malignant progression in oral cancer, in agreement with our results [36, 37].

Enrichment in processes involving actin-filaments and the cytoskeleton were also seen in the genes most informative for separating N, D and T in the PCA. This fits with the largest visible changes in the morphology of these sample types, where disorder in the cellular architecture and orientation, as well as evidence of invasion, are used during histopathological diagnosis. Of interest with the PCA is the almost total separation of N and T but partial overlap of D with both groups (Figure 6). Further longitudinal work is warranted, aimed at similarly clustering non OSCC-associated dysplasia samples in these plots and seeing whether, prospectively, those that group with tumour samples have worse prognosis than those that group with normal oral mucosa. It would then be possible to see whether expression changes of adherens junction components are potential prognostic markers for oral dysplasia.

### Non-coding genes are carcinogenic candidates

Our PCA shows separation of biologically relevant groups is best achieved when ncRNA expression is included (Supplementary Figure S3). We have attempted to highlight non-coding genes that are carcinogenic candidates in a more functionally relevant way using network analysis. Here we have discovered several ncRNAs that are significantly correlated with protein coding genes in small subclusters that are dysregulated at different points in the pathological process. One such cluster is dysregulated early during development of dysplasia i.e. NvD (Figure 7) and independently displays the same enrichment in immune response that the whole list of genes that are DE at this stage exhibit. Within this subcluster are several antisense genes of unknown function, each directly correlating with the protein-coding gene to which is it antisense. Our alignment statistics show high (96 ± 3%, Supplementary Table S2) strand specificity, ensuring these results are not an artifact of reduced strandedness in our data. Also, *RP11-638I2.8* shares no exonic bases with the *WARS* gene to which it is antisense, instilling confidence in the validity of our results. These ncRNAs are candidate regulators of this sub-cluster. Likewise, lincRNA, *RP11-351J23.1* is in a subcluster of genes dysregulated mostly during malignant transformation and so should be further functionally characterized (Supplementary Figure S5).

### Further work

This is the largest gene expression study of its kind, but the nature of the samples (FFPE tissue from surgeries where patients had normal oral epithelium, dysplasia and tumour excised) meant the majority of tissue was required as input for sequencing libraries. This left us unable to systematically validate our findings at the protein level, especially as proteomics studies using FFPE samples are notoriously difficult owing to the cross-linking that occurs during fixation [38]. The hope is that unbiased, systematic studies such as ours highlight new avenues for more focused research into potentially interesting disease mechanisms that are highlighted as occurring at specific disease stages. Examples of this would be a study testing the hypothesis that adherens junctions are diminished in OSCC versus oral dysplasia using tissue microarrays, as has been performed in cervical neoplasias [39].

It is highly desirable to find ways of treating oral dysplasia to either eradicate it, without requiring extensive surgeries that often detrimentally affect the patient's life quality, or prevent its malignant transformation. This study highlights several coding and non-coding genes as candidates in the development and progression of oral dysplasia. Whilst further basic science research is required to validate findings at the protein and/or mechanistic level, we can speculate how our findings may be pursued in the context of translational research.

#### Could oncolytic viruses prevent immune evasion by dysplasia?

Oncolytic viruses target and kill tumour cells, either directly or by triggering a novel or increased host anti-tumour immune response [40, 41]. The latter is potentially relevant to the treatment of oral dysplasia: using oncolytic viruses to boost the host immune response to dysplastic cells, aiming to eradicate them before they can evade the immune system and potentially undergo subsequent malignant transformation. Other immunotherapy approaches could equally be beneficial, but these often include immune checkpoint blockades; oncolytic viruses may be more applicable to oral dysplasia, where malignant transformation is not inevitable, because they are much less toxic and could be applied topically rather than systemically. In support of this, a modified adenovirus, ONYX-015, has previously been applied to oral dysplasia with some success [42]. Our results suggest the potential for further translational research into a) whether immune evasion is part of malignant transformation and, if so, b) the use of oncolytic viruses known to heighten anti-tumour immunity in the treatment of oral dysplasia.

#### Are HOX gene products druggable targets in dysplasia?

The functional activity of HOX gene products requires their association with a co-factor: PBX. This interaction can be blocked by small peptide inhibitor HXR9, leading to global repression of HOX gene function [43]. HRX9 has been shown to induce apoptosis of tumour cells, and reduce tumour growth, when applied to cell-lines derived from breast, ovarian, renal, prostate and non-small-cell lung cancers [43–47]. Our results indicate that upregulation of HOX gene expression is an early event in the development of dysplasia in the oral cavity and, if this result is confirmed at the protein level, application of HOX inhibitors to dysplasia may have therapeutic benefit. This hypothesis can be tested using the commercially available HRX9 molecule.

In conclusion, our results have highlighted several novel mechanisms and specific candidates for further basic scientific work on functional characterization. This includes, but is not limited to, the potential roles of immunoediting, and specifically *IL36G,* and adherens junction components in malignant transformation, and the role of lincRNA *RP11-351J23.1* in de-differentiation of OSCCs.

## MATERIALS AND METHODS

More detail regarding methods, and references to software, are in the Supplementary Document.

### Patient selection

Leeds General Infirmary OSCC patients fulfilling the following criteria were selected and consented: (i) first diagnosis of OSCC, (ii) untreated prior to surgery, (iv) normal and dysplastic epithelium and tumour present in their surgical sample and (v) clinical diagnosis of HPV negative tumour (subsequently confirmed via RNA analysis, see supplemental document). Formalin-fixed paraffin-embedded (FFPE) blocks of surgical sample were the used in this study after a pathologist identified the most representative blocks from which to source each sample type. Patient details are given in Supplementary Table S1. Study ethical codes: REC numbers 07/Q1206/30 and 08/H1306/127.

### RNA extraction

H&E stained FFPE sections underwent an independent, blind, diagnostic pathology review with concurrent marking of specific tissue regions (normal epithelium, dysplastic epithelium and cancer – Figure 1). Non-adjacent areas were selected, and different slides used, for each tissue type wherever possible. Consecutive unstained sections, used for RNA extraction, were heated at 60°C for 3 min, then rehydrated by immersion in xylene for 5 min, 100% ethanol for 5 min, 90% ethanol for 5 min, 70% ethanol for 5 min. Sections were immediately macro-dissected, as per pathologist annotation, using sterile disposable scalpels and tissue placed in a sterile centrifuge tube labelled with patient and sample ID. Total RNA was extracted from macro-dissected tissue using the High Pure FFPE RNA Micro kit according to the manufacturer's instructions (Roche, Burgess Hill, West Sussex, UK) and quantified and quality checked using a Nanodrop™ 8000 (Thermo Fisher scientific Ltd, Altrincham, Cheshire, UK), a 2200 TapeStation (Agilent Technologies UK Ltd, Wokingham, Berkshire, UK) and the Quant-it™ RNA BR Assay kit for the Qubit^®^ 2.0 Fluorometer (Life Technologies Ltd, Paisley, UK).

### Library preparation

Strand-directional whole transcriptome sequencing libraries were prepared using the ScriptSeq™ Complete Kit (Human/Mouse/Rat)-Low Input (Epicentre, Madison, Wisconsin, USA) as per instructions for FFPE samples. The Ampure XP system (BeckmanCoulter) was used for clean-up steps where recommended. Purified Libraries were quality checked and quantified on a 2200 Tapestation using a D1000 screen tape. Samples with >10% adapter contamination were subject to repeat purification using the Ampure XP system.

### Sequencing and alignment

100bp paired-end sequencing was performed on a HiSeq2500. Fastq files were processed using Trim Galore! version 0.2.7, to remove low quality bases, trim adaptors and fix paired-end reads, retaining unpaired reads of at least 35bp post-trimming. Reads were aligned in a strand directional manner to human reference genome GRCh37.p11, using the gencode.v17 genome annotation as a guide, using Tophat2, version 2.0.7. Reads were allowed to align a maximum of five times with maximum two mismatches. Alignment statistics are given in Supplementary Table S2.

### HPV analysis

To confirm HPV status, reads were aligned to the HPV16 and HPV18 genomes using Tophat2. As a positive control we also aligned RNAseq data from a HPV positive patient's trio of samples to these genomes. Zero reads aligned to HPV18 in all cases. The number of reads aligning to HPV16 was zero in all samples from all patients included in this study except PG137 for which there were 7 reads and 4 reads that aligned in the N and T samples respectively. In contrast, the HPV positive patient had zero aligned in their N sample and 4210 and 1137 reads aligning in their D and T samples respectively.

### Expression quantification

Cuffdiff gave Fragments Per Kilobase per million reads Mapped (FPKM) and count data (denoted raw_frags) for each sample, after assigning multireads to their most probably location. Genes with average FPKM ≥ 0.1 across all samples of a tissue type were classed as ‘expressed’ and included in downstream analysis (see Supplemental Document for justification).

### Differential expression analysis

EdgeR was used for differential expression analysis because it can be applied in paired mode. Three pairwise comparisons were made: normal epithelium versus dysplasia (NvD), dysplasia versus tumour (DvT) and normal epithelium versus tumour (NvT). A false discovery rate of 0.01 was used.

### Functional enrichment

The David Bioinformatics Database 6.7 assessed functional enrichment, using all 29,733 expressed genes as the background from which to measure enrichment. Individual lists of significantly differentially expressed genes were input as per the details in the results section, for which the findings are described and tabulated in Figure 2. Gene ontology terms were inspected, and pathway analysis using Biocarta, KEGG and Panther with the latter also used to indicate gene family enrichment. A Benjamini-Hochberg corrected *p*-value <0.05 was significant.

### Heatmaps, boxplots and principal component analysis (PCA)

Heatmaps and boxplots were created in R using the gplots. heatmap.2 and boxplot functions respectively. PCA was performed for (i) all expressed genes, and (ii) expressed protein-coding genes, using the R prcomp function. The gene weightings for the biplot axes that best separated tissue types were acquired from the rotation matrices and averaged to create a ranking, with an R script additionally annotating DE genes (Supplementary Table S3).

### Correlation and network creation

Expressed genes were correlated against one another, as previously described [48]. To ascertain the threshold of significance, 1% of the correlations (∼4.42 × 10^6^ values) were selected at random and the correlation values that demarcated the top and bottom 0.0005% were calculated: −0.74 and 0.95. Correlations equal to or outside these thresholds were retained. The resulting network was plotted using Cytoscape v3.0.2, with annotation files indicating, per gene, the type of gene and whether, and between which pairwise comparisons, it was DE.

### Immune cell analysis

The pathologist (JG) provided a visual estimation of the immune and tumour cell percentage within the annotated/extracted regions of FFPE sections using a light microscope, as is standard practice in clinical diagnosis. Computational quantification was done using the ESTIMATE programme with expressed gene FPKM values for all samples as input. The output was an immune score per sample. Immune-cell specific genes were extracted from the Supplementary Material of Bindea et al [5]. The number of significantly (*p* < = 0.01) upregulated genes of each immune cell type was compared to the total number of significantly upregulated genes, in the NvD and DvT analyses separately. A Fisher's test (*p* < = 0.05) was used to identify immune cell types for which a significant number of genes had been upregulated in either analysis. The CYT scores were calculated as the geometric mean (log_2_ of the average) of expression, in FPKM, of *GZMA* and *PRF1* as devised in [7].

### Independent dataset validation

We used two datasets to validate our findings, where possible. GSE30784 consists of Affymetrix U133 2.0 Plus GeneChip microarray expression data from 45 normal oral samples, 17 oral dysplasias and 167 oral carcinomas (HPV status not indicated) from different individuals [33]. The quality of data from each microarray was assessed, and confirmed, by downloading the raw .CEL file data from the Gene Expression Omnibus (GEO) and running the AffyQCReport package in R. Expression values, normalized and background corrected using the gcRMA package in R, were then downloaded from the Gene Expression Omnibus (GEO) and collapsed into gene expression values using the collapseRows package in R with ‘MaxMean’ function [49]. Pairwise differential gene expression analysis was performed using the limma package with an adjusted *p*-value significance threshold of 0.05. Fisher tests were then used to compare the number of DEGs in each immune cell category, according to the Bindea et al [5] classification, in the NvD and DvT comparisons. GSE26549 consists of Affymetrix Human Gene 1.0 ST microarray expression from 86 patients with oral dysplasia, of which 35 developed oral cancer over a median follow up of 6.08 years [23]. We refer to the two groups within these data as dysplasias that progressed and those that did not. The accompanying publication for this dataset included lists of genes that are DE between the two groups, and their direction of dysregulation. These lists were downloaded and functional enrichment in different subsets were evaluated using ToppFun [50].

## SUPPLEMENTARY RESULTS




