# Supplementary material for: Elucidating drivers of oral epithelial dysplasia formation and malignant transformation to cancer using RNAseq
Source: Oncotarget. 2015 Oct 19;6(37):40186–201. doi: 10.18632/oncotarget.5529 (PMC4741888; doi:10.18632/oncotarget.5529)
Supplement: Supplementary file 1 [file oncotarget-06-40186-s001.pdf]

SUPPLEMENTARY RESULTS

Clinical information for the 19 HPV-negative patients used in this study is given in Supplementary Table S1. Each patient had three samples sequenced: normal oral mucosa (N), dysplasia (D) and OSCC tumour (T). On average 490ng total RNA was used per sample resulting in an average library output of weight: 120ng and fragment length: 200bp. Each library passed quality assessment via the bioanalyser and, following processing, every sequencing file (fastq) passed the per-base and per-sequence quality tests in the FastQC programme. Sequencing resulted, on average, in 79 ± 33 million reads, with 76% ± 8% aligning, per sample (Supplementary Table S2). These metrics indicate that the RNA extracted from FFPE tissue was sufficient for high-quality sequencing results; highly-degraded RNA would produce adapter-contaminated libraries (evident on bioanalyser traces) and not result in such high percentages of unique alignments. Gene expression was quantified as Fragments per Kilobase per million Mapped (FPKM) for each of 62,766 protein-coding and non-coding genes. In total 29,733 of these were shown to be expressed (average FPKM across any of the three groups: N, D or T, of at least 0.1 FPKM) and used in downstream analysis.

SUPPLEMENTARY METHODS

Study design and patient selection

Patients were selected from an existing prospective sample collection from patients attending the Oral surgery outpatients' clinic of Leeds General Infirmary with potential or proven cancerous oral lesions. The study was approved by the local ethics committee; (REC ref no 07/Q1206/30 and 08/H1306/127). All patients provided informed consent and were anonymised. All formalin-fixed paraffin-embedded (FFPE) blocks from the patient's surgical sample were then obtained from archive to produce the research samples used in this study.

Criteria for patient selection were as follows: patients had (i) OSCC, (ii) no previous OSCC (iii) no adjuvant treatment prior to surgery, (iv) tumour, normal epithelium and dysplastic epithelium present in their surgical sample and (v) clinical diagnosis of HPV negative tumour (subsequently confirmed via RNA analysis, see below), were selected for further sampling (see Supplementary Table S1). All blocks derived from the surgical samples were then reviewed by a pathologist to identify the most representative blocks from which to source each sample type for every patient. Care was taken to select non-adjacent areas for each sample type wherever possible.

RNA extraction from FFPE samples

Total RNA was prepared from the above selected samples from macro-dissected FFPE tissue using a commercially available kit. Briefly, 4 µm-thick sections were cut from each selected FFPE tissue block and stained with H&E. An independent pathologist, blind to the patient identity and diagnosis, reviewed all the marked H&E slides in order to (i) confirm the diagnosis and histology reported in the original pathology report; (ii) mark representative areas of normal epithelium, dysplastic epithelium and cancer for macro-dissection and (iii) evaluate and record the percentage of tumour cells in the marked area (Figure 1). Seven further consecutive 10 µm-thick sections were cut from each block for RNA extraction, followed by a final 4 um slide for H&E staining and review by the pathologist for persistence of histology throughout the sampling. Slides for RNA extraction were heated on a hot plate at 60°C for 3 min, and then rehydrated by immersion in xylene for 5 min, 100% ethanol for 5 min, 90% ethanol for 5 min, 70% ethanol for 5 min. Sections were immediately macro-dissected using sterile disposable scalpels to harvest the desired tissue area; the corresponding H&E stained, marked slide was used as a guide and great care was taken to avoid sampling adjacent areas of tissue. All the macro-dissected tissue from each individual sample was placed in a separate sterile centrifuge tube labelled with the unique patient study ID and sample ID. RNA extraction was performed using the High Pure FFPE RNA Micro kit according to the manufacturer's instructions (Roche, Burgess Hill, West Sussex, UK). RNA samples were quantified and quality checked using a Nanodrop™ 8000 (Thermo Fisher scientific Ltd, Altrincham, Cheshire, UK), a 2200 TapeStation (Agilent Technologies UK Ltd, Wokingham, Berkshire, UK) and the Quant-it™ RNA BR Assay kit for the Qubit® 2.0 Fluorometer (Life Technologies Ltd, Paisley, UK).

Library preparation

Strand-directional whole transcriptome sequencing libraries were prepared using the ScriptSeq™ Complete Kit (Human/Mouse/Rat)-Low Input (Epicentre, Madison, Wisconsin, USA), following the manufacturers instructions for FFPE samples using the Ampure XP system (BeckmanCoulter) for clean-up steps where recommended. Briefly, total RNA samples were treated with Ribo-zero to remove rRNA, followed by a column clean-up and quality check on a 2200 Tapestation using an R6K high sensitivity screen tape. rRNA depleted samples were then subject to fragmentation, cDNA synthesis and terminal tagging prior to PCR amplification and index tagging

using the ScriptSeq™ Index PCR primers (epicentre). Purified Libraries were quality checked and quantified on a 2200 Tapestation using a D1000 screen tape. Samples with >10% adapter contamination were subject to repeat purification using the Ampure XP system.

Sequencing and alignment

100bp paired-end sequencing was performed on a HiSeq2500. Samples were barcoded and sequenced to the equivalent of four samples per lane by sequencing 12 samples over three lanes, where possible, to alleviate possible lane effects. After merging data for the same samples from different lanes, the result was two fastq files, one for each paired end read, per sample. Fastq files were processed using Trim Galore! version 0.2.7, to remove low quality bases, trim adaptors and fix paired-end reads, retaining unpaired reads of at least 35bp post-trimming (http://www.bioinformatics.babraham.ac.uk/projects/trim_galore/). At this stage there are three fastq files per sample: one for each paired read and one containing unpaired reads. Two alignments are then performed per sample, a paired end alignment and single end alignment of the unpaired reads. Each alignment uses Tophat2, version 2.0.7, to align reads in a strand directional manner to human reference genome GRCh37.p11, using the gencode.v17 genome annotation as a guide, allowing each read to align a maximum of five times (1). Two mismatches were allowed per read, with only one in the first 20bp. The two alignment files were merged, sorted and reheaded to create a single bam file per sample. An example of the commands used, detailing each parameter, are:

Trim_galore

~/bin/trim_galore_zip/trim_galore —dont_gzip -t —paired —fastqc —retain_unpaired -a AGATCGGAAGAGC -o OSCC_Sample/OSCC_Sample_R1.fastq.gz OSCC_Sample_R2.fastq.gz

Tophat2

tophat2 -g 5 -i 30 —no-coverage-search —microexon-search —read-realign-edit-dist 0 —b2-N 1 —library-type fr-secondstrand —transcriptome-index gencode.v17.chr_patch_hapl_scaff.TRANSCRIPTOME -x 20 -M -o OSCC_Sample/ GRCh37.p11.genome OSCC_Sample/processed_read1.fq OSCC_Sample/processed_read2.fq

Alignment statistics

The alignment statistics, detailed in Supplementary Table S2 were ascertained using the samtools flagstat command, applied to each bam file. The number of unique and multireads is assessed according to the NH:i tag, indicating the number of alignments per read, in the bam files. The percentage of reads aligning to various features (introns, ribosomal RNA, mRNA, intergenic regions) and to the correct strand are ascertained using the CollectRnaSeqMetrics programme in the Picard software suite, version 1.56 (http://picard.sourceforge.net). An example command for CollectRnaSeqMetrics is:

java -Xmx4G -jar ~bin/picard-tools-1/picard-tools-1.56/CollectRnaSeqMetrics.jar REF_FLAT= hg19_refFlat.txt RIBOSOMAL_INTERVALS= RibosomeIntervals.txt STRAND_SPECIFICITY= FIRST_READ_TRANSCRIPTION_STRAND MINIMUM_LENGTH = 50 CHART_OUTPUT= OSCC_Sample.pdf INPUT= OSCC_Sample.bam OUTPUT= OSCC_Sample.txt VALIDATION_STRINGENCY=SILENT

HPV analysis

To confirm that our samples were HPV negative, as suggested by the clinical reports, we aligned all sequenced reads to the HPV16 and HPV18 genomes using Tophat2. As a positive control we also aligned RNAseq data from a HPV positive patient's trio of samples to these genomes. The number of RNA derived reads aligning to the HPV18 genome was zero in all cases. The number of RNA derived reads aligning to the HPV16 genome was zero in all samples from all patients included in this study except PG137 for which there were 7 reads and 4 reads that aligned in the N and T samples respectively. In contrast, the HPV positive patient had zero aligned in their N sample and 4210 and 1137 reads aligning in their D and T samples respectively.

Expression quantification

Bam files were input to cuffdiff, version 2.1.1, in patient matched pairs (2). Cuffdiff is used to assign multireads (reads that have more than valid alignment) to a single location using the –u parameter. The cuffdiff output, used for downstream analysis, was the Fragments Per Kilobase per million reads Mapped (FPKM) and the count data (denoted raw_frags) required by the edgeR programme. FPKM is the normalised expression metric used to compare genes within and between samples. A threshold of 0.1 was selected to identify 'expressed' genes as this was the value of the 1st quantile in a boxplot of all non-zero FPKM values for all genes in all samples. Only genes with an average FPKM of 0.1 across all samples belonging to a group (N: normal mucosal epithelium, D: dysplasia or T: tumour) were included in the downstream analysis. An example CuffDiff command is:

cuffdiff -u —library-type fr-secondstrand —max-bundle-frags 5000000 —FDR 0.1 -L N,D -o outDir/OSCC_Sample_NvD/ gencode.v19.chr_patch_hapl_scaff.annotation.gtf OSCC_Sample_N.bam OSCC_Sample_D.bam

Differential expression analysis

EdgeR was used (3), as opposed to using cuffdiff directly, for differential expression analysis because it can be applied in paired mode using the generalized linear modeling approach as demonstrated in section 4.4 of the edgeR User's Guide (March 2013). Three pairwise comparisons were made: normal epithelium versus dysplasia (NvD), dysplasia versus tumour (DvT) and normal epithelium versus tumour (NvT). A false discovery rate of 0.01 is used to determine differential expression. This is an example script used in the edgeR analysis, complete with comments:

Library(edgeR)

#The raw data is the rounded count, following multi-read assignment, stored in the genes.read_group_tracking output file from Cuffdiff (column is raw_frags). These values have been amalgamated for each patient for both N and D samples and stored in the data file edgeR_genes.counts

edgeR_genes.counts.

```
raw.data < -read.delim("edgeR_NvD_genes/edgeR_genes.counts", header=T)
```

#The fpkm data also present in the genes.read_group_tracking output file from Cuffdiff (column is FPKM). These values have been amalgamated for each patient for both N and D samples and stored in the data file edgeR_genes.fpkm

edgeR_genes.fpkm

```
fpkm.data < -read.delimedgeR_NvD_genes/edgeR_genes.fpkm", header=T)


Group < -factor(c(rep("N",19),rep("D",19)))
Patient < -factor(c("PG004","PG038","PG049" "PG079","PG129","PG086","PG105","PG108" "PG114","PG122","PG123","PG136","PG137" "PG144", "PG146","PG174","PG187","PG192", "PG063","PG004","PG038","PG049","PG079","PG129","PG086","PG105","PG108","PG114", "PG122","PG123","PG136","PG137","PG144","PG146","PG174","PG187","PG192","PG063"))
```

#Ensure a matched sample analysis

```
Design < -model.matrix(~Patient+Group)

dgefunction < -function(df,design,name)
{
y < -DGEList(counts=df[,4:ncol(raw.data)],genes=df[,1:3])
y < -calcNormFactors(y)
rownames(design) < -colnames(y)
y < -estimateGLMCommonDisp(y,design)
y < -estimateGLMTrendedDisp(y,design)
y < -estimateGLMTagwiseDisp(y,design)
fit < -glmFit(y,design)
lrt < -glmLRT(fit)
```

#Add multiple testing corrections

```
lrt$table$p.adj < -p.adjust(lrt$table$PValue,method="BH")
res < -merge(lrt$genes,lrt$table,by = 0)
res$ http://Row.names < -NULL
```

#edgeR outputs read counts used in the analysis as Counts Per Million (CPM)

```
count < -as.data.frame(cpm(y))
count$Genes < -lrt$genes$GeneID
addcpm < -merge(res,count,by.x="GeneID",by.y="Genes")
```

#Ensure the final table includes the count and FPKM value for each gene, alongside its common name, as well as the stats that indicate significance

```
results < -merge(addcpm,fpkm.data,by.x="GeneID",by.y="EnsID")
write.table(results,file=paste(name),sep="\t",quote=F,
http://row.names=F)
}
dgefunction(raw.data,Design,"edgeR_NvD_genes/NvD_allGeneTypes_DGE.txt")
```

Functional enrichment

The David Bioinformatics Database 6.7 was used to assess functional enrichment, via the web server (4). The background from which to measure enrichment was the list of all genes expressed within at least one of the sample groups (29,733 genes). These were input using their Ensembl ID. Individual gene lists were then input as per the details in the results section, for which the findings are described and tabulated in Figure 2. Gene ontology terms were inspected, and pathway analysis using Biocarta, KEGG and Panther. Gene family enrichment was also inspected based on the Panther database terminology. Significant associations were those with a *p*-value < 0.05 following a Benjamini-Hochberg multiple testing correction.

Heatmaps, boxplots and principal component analysis (PCA)

Heatmaps were created from $\log_2$ fold change values in R using the heatmap.2 function in the gplots library. Colours were selected from the colorRampPalette in the RColorBrewer library. Boxplots were created in R using the standard boxplot function. PCA was performed for all expressed genes, and separately for all expressed protein-coding genes, using the prcomp function in R. An iterative R script was used to plot the first ten PCs against one another, with the group (N, D or T) that the sample belonged to annotated. Upon determining the biplot that best separated the three biologically relevant groups, the weighting of each gene within those PCs was ascertained by accessing the relevant rotation matrix for the prcomp object in question. The average magnitude of the weights given to each gene by the two most informative PCs was

used to rank the genes in this analysis and an R script was used to annotate each gene according to whether it had was DE in any of the pairwise comparisons.

Correlation and network creation

All 29,733 expressed genes were correlated against one another, as previously described (5). Briefly, the correlation matrix was generated by multiplying the input expression matrix, after normalizing to give each gene a mean of 0 and a magnitude of 1, by its transpose. To ascertain the threshold of significance, 1% of the correlations (~4.42×10^6 values) were selected at random and the correlation values that demarcated the top and bottom 0.0005% were calculated: −0.74 and 0.95. Any correlations equal to or below or above these thresholds, respectively, were retained. The resulting network was plotted using Cytoscape v3.0.2, with annotation files indicating, per gene, the type of gene and whether, and between which pairwise comparisons, it was DE.

Immune cell quantification

ESTIMATE was run using the expressed gene FPKM values for all samples as input. The output was an immune score and stroma score per sample. Score differences were then calculated for each patient as score Y minus score X for an XvY analysis. Statistical tests were run in R, using Shapiro-Wilk tests to confirm the data were normally distributed before performing paired *t*-tests.

Immune-cell specific genes were extracted from the supplemental material of Bindea et al (6). The number of significantly ($p < = 0.1$) upregulated genes of each immune cell type was compared to the total number of significantly upregulated genes, in the NvD and DvT analyses separately. A Fisher's test ($p <=0.05$) was used to identify immune cell types for which a significant number of genes had been upregulated in either analysis.

REFERENCES

1. Kim D, Pertea G, Trapnell C, Pimentel H, Kelley R, Salzberg S. TopHat2: accurate alignment of transcriptomes in the presence of insertions, deletions and gene fusions. Genome Biology. 2013; 14:R36.
2. Trapnell C, Hendrickson DG, Sauvageau M, Goff L, Rinn JL, Pachter L. Differential analysis of gene regulation at transcript resolution with RNA-seq. Nat Biotech. 2013; 31:46–53.
3. Robinson MD, McCarthy DJ, Smyth GK. edgeR: a Bioconductor package for differential expression analysis of digital gene expression data. Bioinformatics. 2010; 26:139–40.
4. Huang DW, Sherman BT, Lempicki RA. Systematic and integrative analysis of large gene lists using DAVID bioinformatics resources. Nat Protocols. 2008; 4:44–57.
5. Rane Jayant K, Droop Alastair P, Pellacani D, Polson Euan S, Simms Matthew S, Collins Anne T, et al. Conserved Two-Step Regulatory Mechanism of Human Epithelial Differentiation. Stem Cell Reports. 2:180–88.
6. Bindea G, Mlecnik B, Tosolini M, Kirilovsky A, Waldner M, Obenauf Anna C, et al. Spatiotemporal Dynamics of Intratumoral Immune Cells Reveal the Immune Landscape in Human Cancer. Immunity. 2013; 39:782–95.

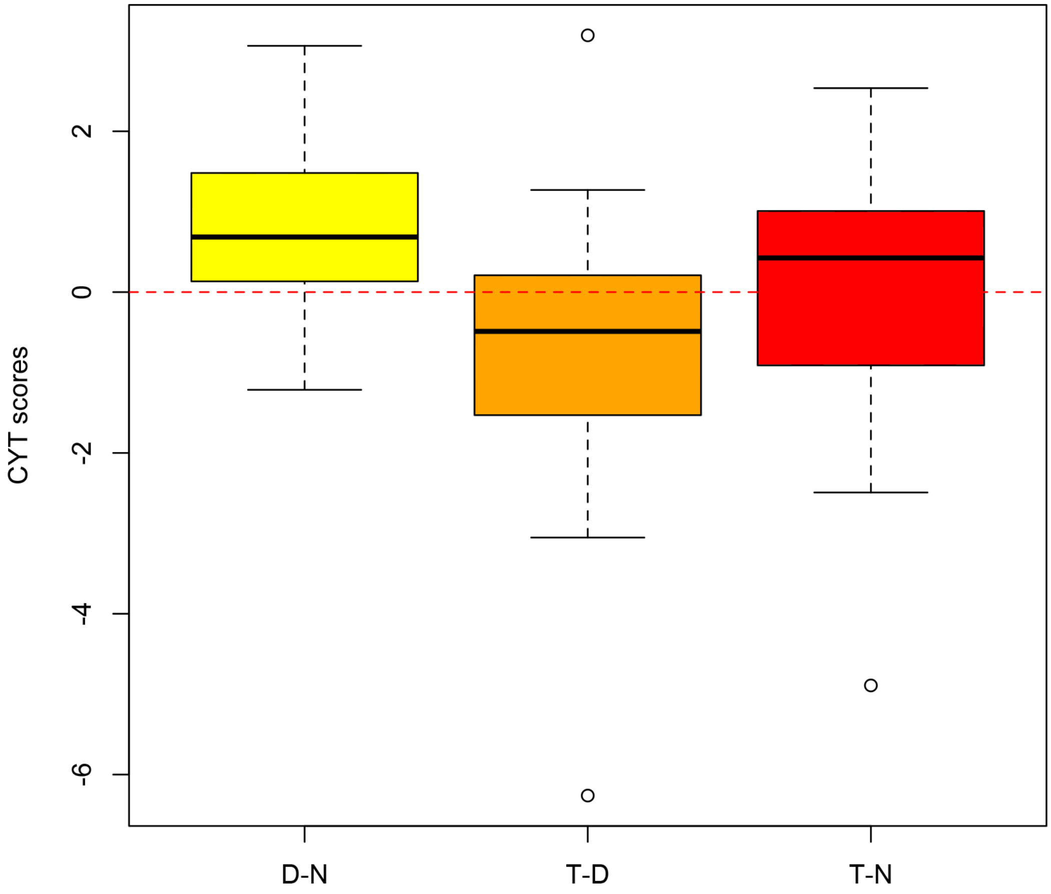

Supplementary Figure S1: Boxplots showing the change in cytolitic activity, as scored via the CYT metric (geometric average of *GZMA* and *PRF1* expression as per [7]) between matched samples. D-T Dysplasia score minus Normal score. T-D: Tumour score minus Dysplasia score. T-N Tumour score minus Normal score.

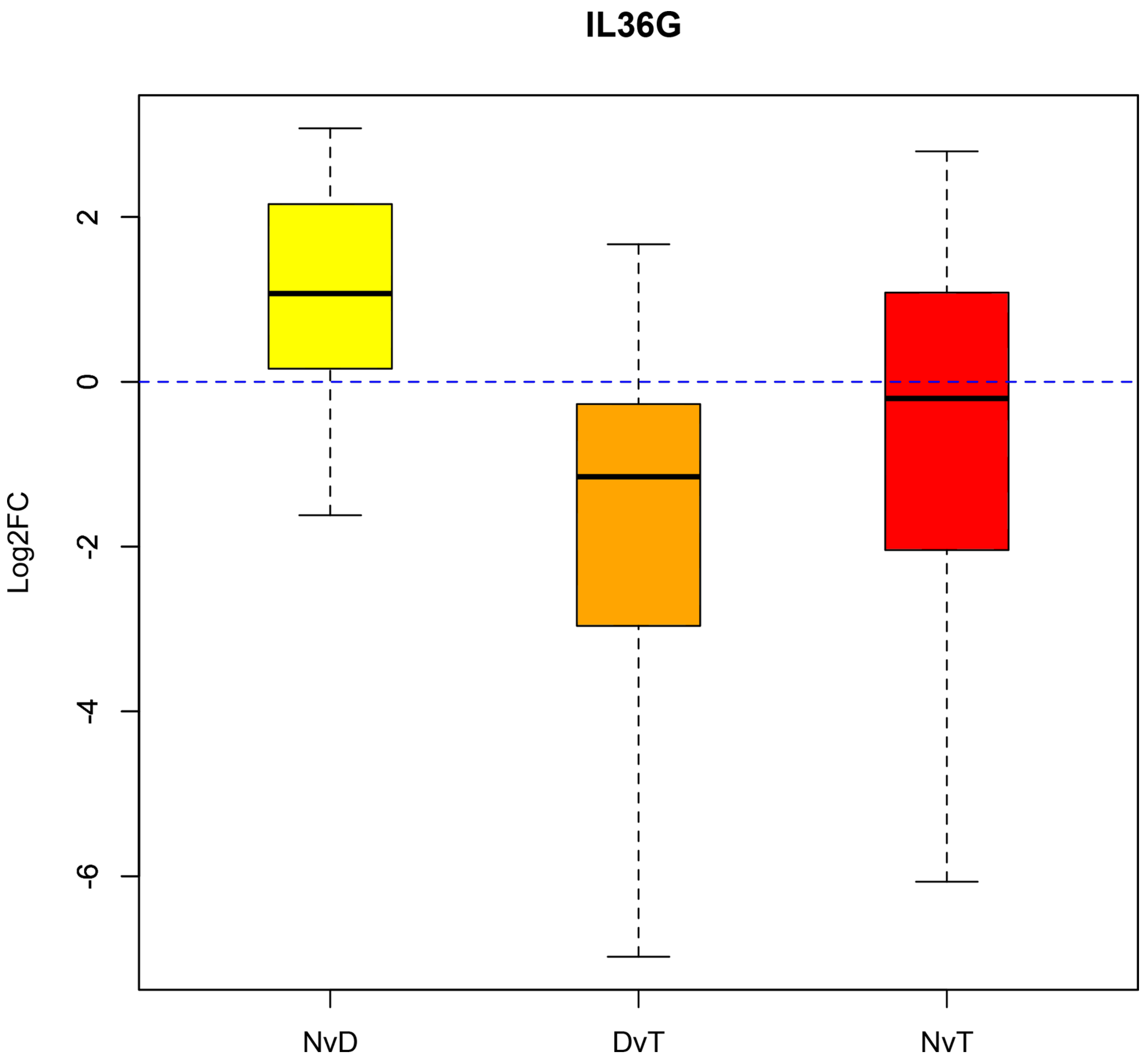

Supplementary Figure S2: Boxplots showing the distributions of log_2 fold change (Log2FC) values for Interleukin 36 gamma (*IL36G*) for all 19 samples in our data for the different pairwise comparisons. NvD: Normal versus Dysplasia. DvT: Dysplasia versus Tumour. NvT: Normal versus Tumour.

A

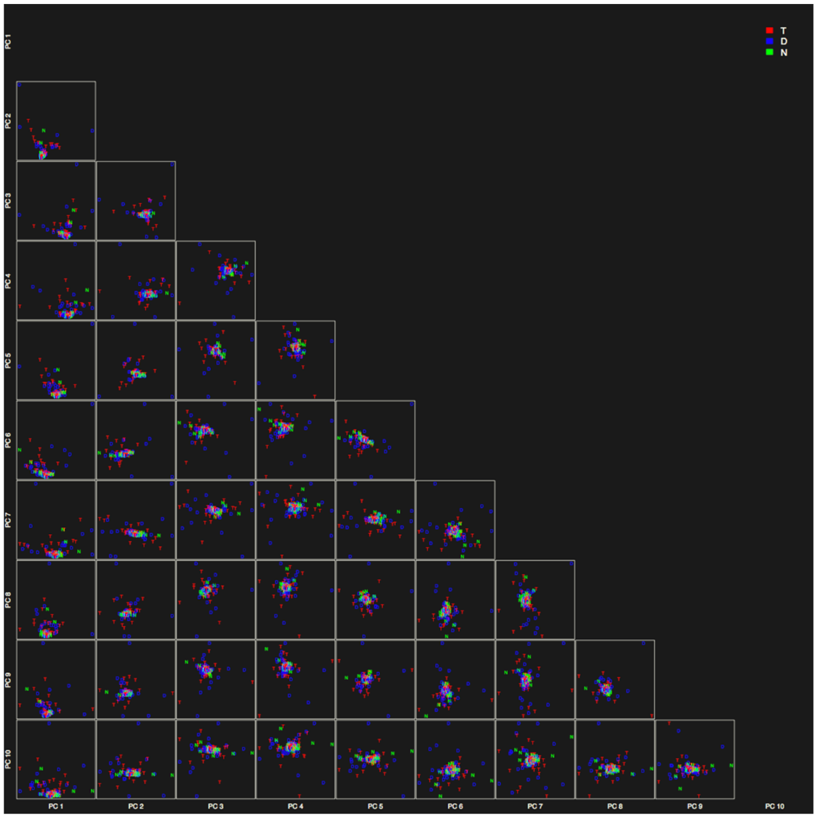

B

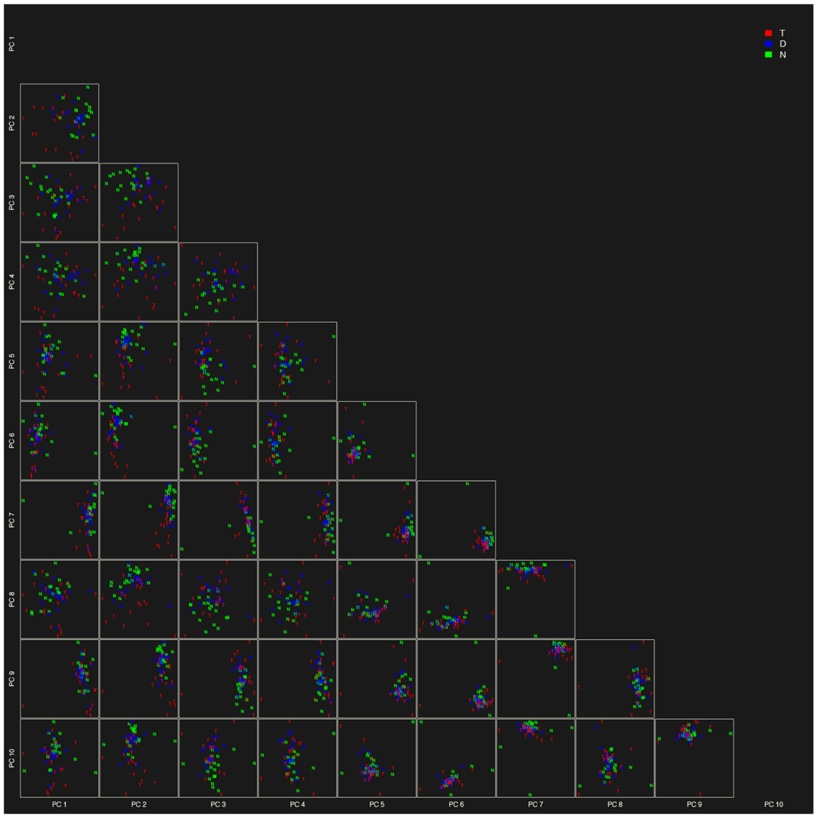

Supplementary Figure S3: All possible principal component (PC) biplots for PCs 1 to 10 for analysis that includes. A. only protein-coding genes, or B. all genes. Red T symbols indicate tumour, blue D symbols indicate dysplasia and green N symbols indicate normal samples. The matched nature of the trios is not used in this analysis. Higher resolution copies of these images can be downloaded from www.bioinformatics.leeds.ac.uk/~bs06lw/SteadSupplementalFigs/.

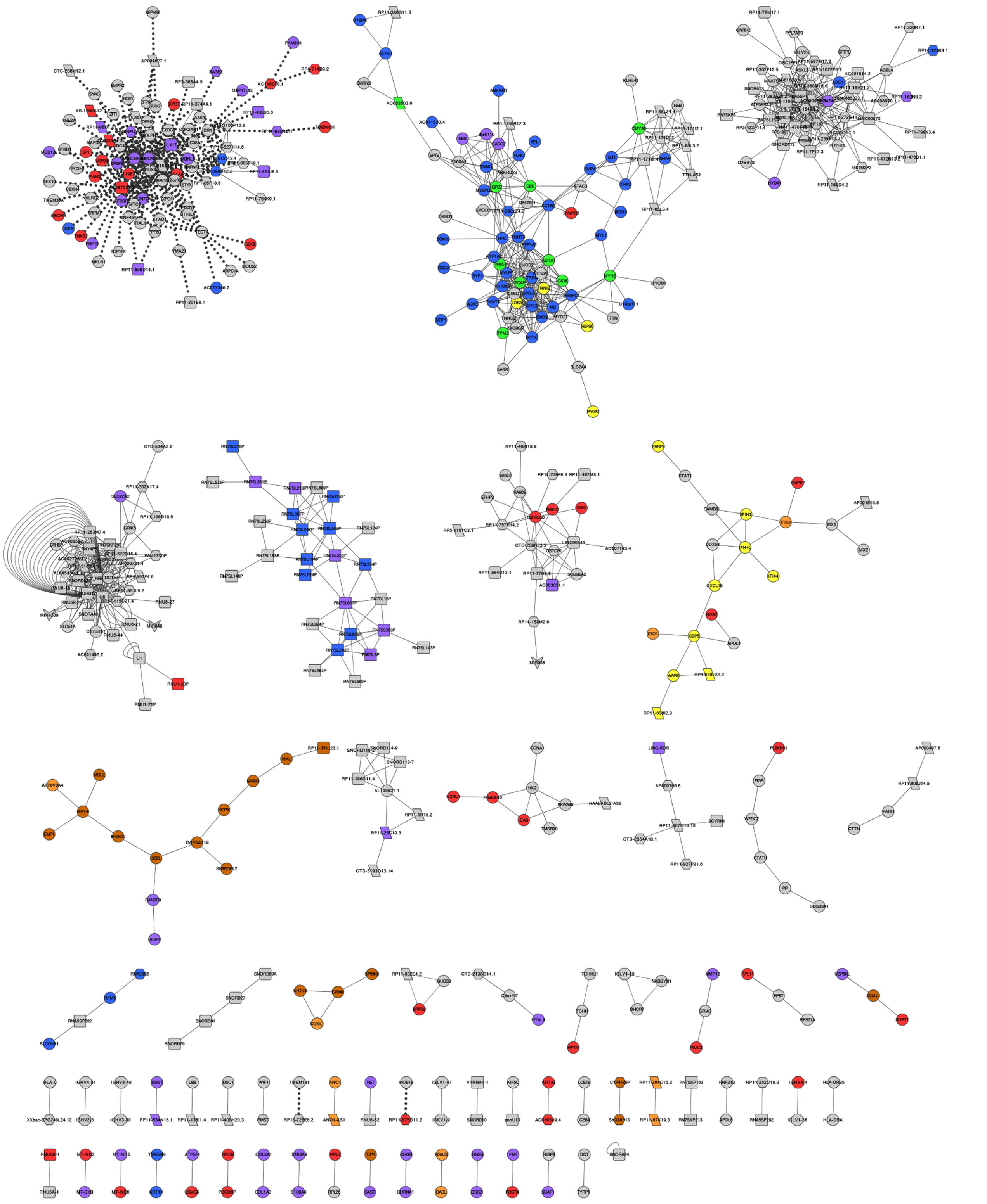

Supplementary Figure S4: Genes with significantly (0.001th percentile) correlated expression across all 57 samples in our study. Each node is a gene, with the name annotated. The shape indicates the type of gene, and the colour indicates whether the gene has been found differentially expressed in one or more pairwise comparisons of our data (more detailed key given below). A higher resolution copy of this image can be downloaded from www.bioinformatics.leeds.ac.uk/~bs06lw/SteadSupplementalFigs/.

Colour key (DE genes in the following comparisons):

Yellow – NvD
Orange – NvD and NvT
Green – NvD and DvT
Blue – DvT
Purple – DvT and NvT
Red – NvT
Brown – NvD, DvT and NvT
Gray – Not DE in any comparison

Shape key:

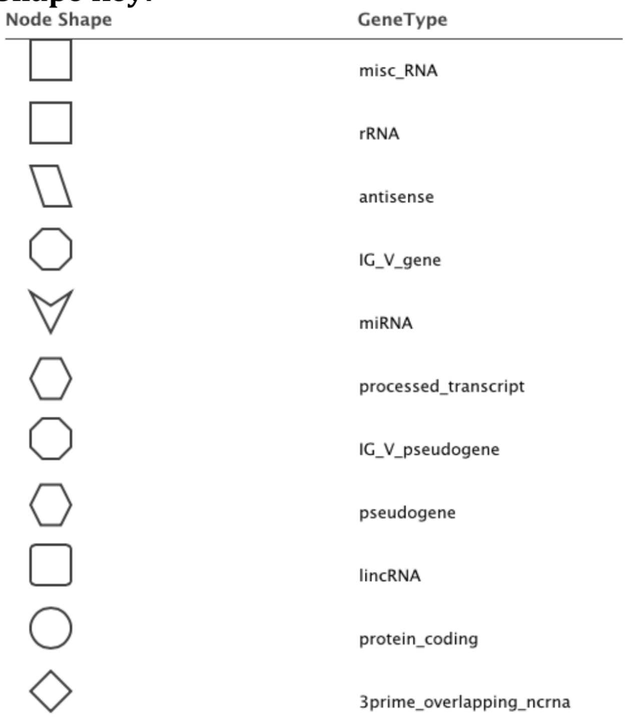

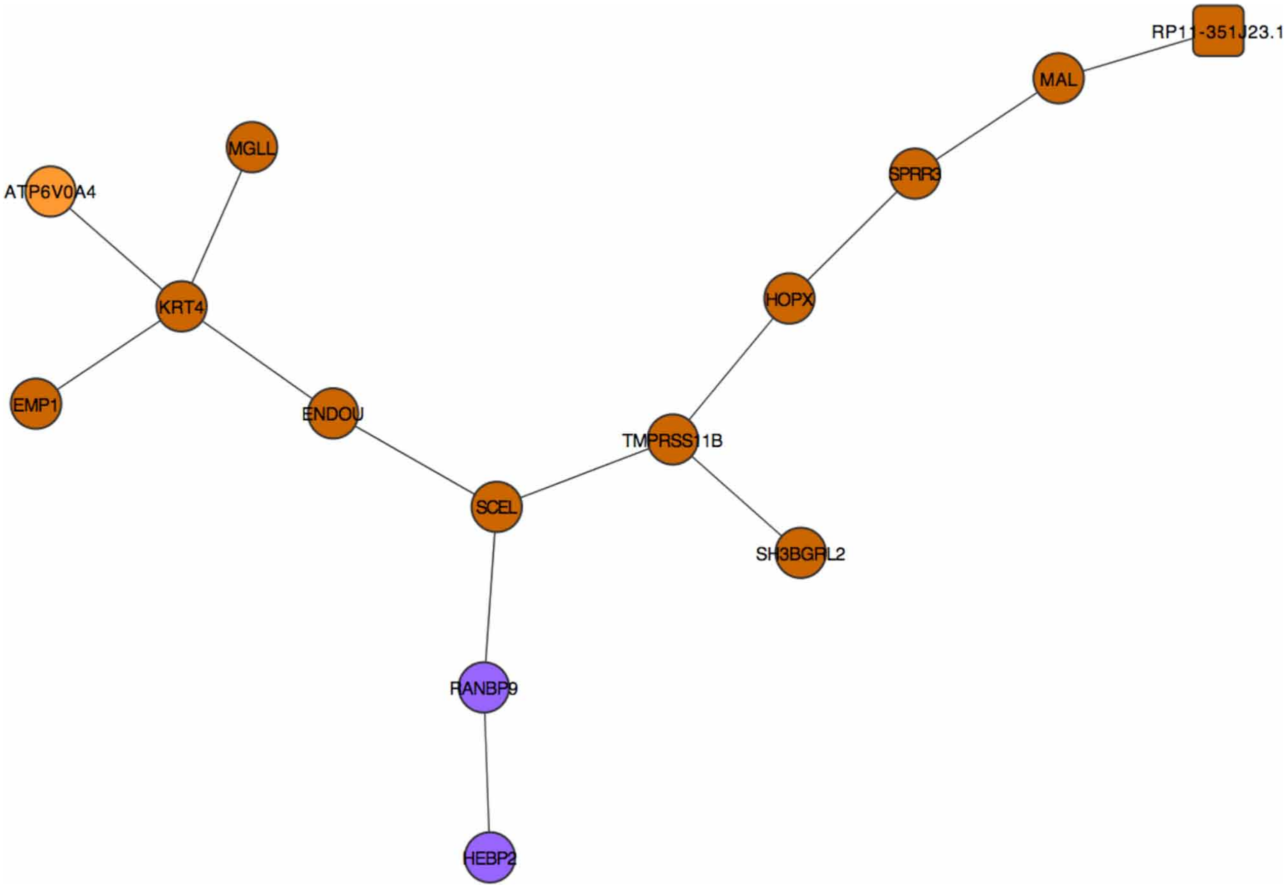

Supplementary Figure S5: A subcluster of significantly positively correlated genes. See the key accompanying Supplementary Figure S4.

Supplementary Table S1: Clinical information for the 19 patients used in this study.
(*These data are accessed from a tab of the Supplementary Tables spreadsheet.*)

Supplementary Table S2: Alignment statistics for all 57 samples sequenced in this study (19 patients, with a trio of samples per patient). N: normal oral mucosa, D: oral dysplasia, T: oral squamous cell tumour.
(*These data are accessed from a tab of the Supplementary Tables spreadsheet.*)

Supplementary Table S3: The log2 fold change (Log2FC) and Benjamini-Hochberg corrected *p*-value (p.adj) for each pairwise comparison in our analyses.
(*These data are accessed from a tab of the Supplementary Tables spreadsheet.*)

Supplementary Table S4: The number of differentially expressed genes (DEGs) per transcript subtype and pairwise comparison

Transcript Type	No. DEGs	NvD	DvT	NvT
**protein-coding**	Total	429	1370	2977
	Up	197	425	801
	Down	232	945	2176
**lincRNA**	Total	63	113	254
	Up	41	60	127
	Down	22	53	127
**antisense**	Total	48	214	367
	Up	18	41	78
	Down	30	173	289
**pseudogene**	Total	29	140	231
	Up	14	20	50
	Down	15	120	181
**Other**	Total	32	170	282
	Up	26	62	83
	Down	6	108	199
**TOTAL**	Total	601	2007	4111
	Up	296	608	1139
	Down	305	1399	2972

NvD: Normal versus Dysplasia. DvT: Dysplasia versus Tumour. NvT: Normal versus Tumour.

Supplementary Table S5: Per sample immune cell estimates from pathologist or Estimation of STromal and Immune cells in MAlignant Tumours using Expression data (ESTIMATE) programme

Patient ID	Pathologist Immune Cell % Estimate		ESTIMATE programme ImmuneScore	
	Dysplasia	Tumour (OSCC)	Dysplasia	Tumour (OSCC)
**PG004**	5	5	−244.3822949	64.94964718
**PG038**	10	60	495.725657	603.1471249
**PG049**	15	15	417.8335924	−633.5966277
**PG063**	15	40	675.3443393	697.560965
**PG079**	15	30	1295.173942	943.8604289
**PG086**	3	30	−58.97729651	1507.266758
**PG105**	10	15	214.0683206	1242.433871
**PG108**	2	15	685.8216549	375.9206624
**PG114**	1	30	113.1191602	387.4684445
**PD122**	20	5	908.913445	399.6396517
**PG123**	30	5	1180.281587	165.785118
**PG129**	70	10	1853.812302	1755.092716
**PG136**	20	50	1530.36249	1633.054984
**PG137**	10	5	881.611769	214.9216487
**PG144**	10	7	28.98644522	47.05302571
**PG146**	40	40	1674.244237	1800.339055
**PG174**	10	10	−171.8035166	−28.01364698
**PG187**	40	25	599.2821744	495.424031
**PG192**	15	10	492.4604704	-516.2939351

Supplementary Table S6: Immune cell types that have a significant number of genes DE within the unmatched validation cohort (GSE30784), and the specific genes regulated in a certain direction therein

NvD			DvT		
**Immune Type***	**Gene Name**	**Direction**	**Immune Type***	**Gene Name**	**Direction**
iDC	PREP	Upregulated	Macrophages	RAI14	Upregulated
	SLC7A8	Upregulated		APOE	Upregulated
	CTNS	Upregulated		CXCL5	Upregulated
	SLC26A6	Upregulated		CD163	Upregulated
	VASH1	Upregulated		CYBB	Upregulated
	MMP12	Upregulated		BCAT1	Upregulated
	CD1B	Upregulated		SCG5	Upregulated
Mast cells	SLC18A2	Upregulated		MS4A4A	Upregulated
	PTGS1	Upregulated		MSR1	Upregulated
	HDC	Upregulated		CHI3L1	Upregulated
	ABCC4	Upregulated		CD84	Upregulated
	TPSAB1	Upregulated		FN1	Upregulated
	TPSB2	Upregulated	Mast cells	MS4A2	Downregulated
	SCG2	Upregulated		CMA1	Downregulated
	MS4A2	Upregulated		CTSG	Downregulated
	CPA3	Upregulated		TAL1	Downregulated
				SLC24A3	Downregulated
				PTGS1	Downregulated
				HDC	Downregulated
				SLC18A2	Downregulated
				GATA2	Downregulated
				HPGD	Downregulated
				NR0B1	Downregulated

* According to Bindea et al [5].
NvD: Normal versus Dysplasia. DvT: Dysplasia versus Tumour. iDC: immature Dendritic Cell

Supplementary Table S7: Genes that are significantly differentially expressed in all three pairwise comparisons.
(*These data are accessed from a tab of the Supplementary Tables spreadsheet.*)

Supplementary Table S8: The weighting given to each gene in the principal components (PCs) that best separate our data into biologically relevant sample groups.
(*These data are accessed from a tab of the Supplementary Tables spreadsheet.*)

Supplementary Table S9: Information on the members of a subcluster of significantly correlated genes in our dataset (Figure 5), taken from online gene information databases.
(*These data are accessed from a tab of the Supplementary Tables spreadsheet.*)

Supplementary Table S10: Genes upregulated in non-progressive versus progressive dysplasia (reference 19) and in stimulated versus naïve T-cells (MSigDB C7: Nick Haining Lab [DFCI])

Entrez Gene ID	Gene Symbol	Gene Name
53373	TPCN1	two pore segment channel 1
7375	USP4	ubiquitin specific peptidase 4 (proto-oncogene)
8440	NCK2	NCK adaptor protein 2
10493	VAT1	vesicle amine transport 1
275	AMT	aminomethyltransferase
5412	UBL3	ubiquitin-like 3
6609	SMPD1	sphingomyelin phosphodiesterase 1, acid lysosomal
8665	EIF3F	eukaryotic translation initiation factor 3, subunit F
65018	PINK1	PTEN induced putative kinase 1
3590	IL11RA	interleukin 11 receptor, alpha
54861	SNRK	SNF related kinase
9812	KIAA0141	KIAA0141
677	ZFP36L1	ZFP36 ring finger protein-like 1
50854	C6orf48	chromosome 6 open reading frame 48
53947	A4GALT	alpha 1,4-galactosyltransferase
10961	ERP29	endoplasmic reticulum protein 29
2811	GP1BA	glycoprotein Ib (platelet), alpha polypeptide
23353	SUN1	Sad1 and UNC84 domain containing 1
9138	ARHGEF1	Rho guanine nucleotide exchange factor (GEF) 1
55268	ECHDC2	enoyl CoA hydratase domain containing 2
5094	PCBP2	poly(rC) binding protein 2
6122	RPL3	ribosomal protein L3
